# Enteroinsular axis response to carbohydrates and fasting in healthy newborn foals

**DOI:** 10.1111/jvim.15641

**Published:** 2019-10-30

**Authors:** Lindsey M. Rings, Jacob M. Swink, Laura K. Dunbar, Teresa A. Burns, Ramiro E. Toribio

**Affiliations:** ^1^ College of Veterinary Medicine The Ohio State University Columbus Ohio

**Keywords:** equine, incretin, insulin, intestine, neonate, pancreas

## Abstract

**Background:**

The enteroinsular axis (EIA) comprises intestinal factors (incretins) that stimulate insulin release after PO ingestion of nutrients. Glucose‐dependent insulinotropic polypeptide (GIP) and glucagon‐like peptide‐1 (GLP‐1) are the main incretins. The EIA has not been investigated in healthy neonatal foals but should be important because energy demands are high in healthy foals and dysregulation is frequent in sick foals.

**Objectives and Hypothesis:**

To evaluate the EIA response to carbohydrates or fasting in newborn foals. We hypothesized that incretin secretion would be higher after PO versus IV carbohydrate administration or fasting.

**Animals:**

Thirty‐six healthy Standardbred foals ≤4 days of age.

**Methods:**

Prospective study. Blood was collected before and after a PO glucose test (OGT; 300, 500, 1000 mg/kg), an IV glucose test (IVGT; 300, 500, 1000 mg/kg), a PO lactose test (OLT; 1000 mg/kg), and fasting. Foals were muzzled for 240 minutes. Blood was collected over 210 minutes glucose, insulin, GIP, and GLP‐1 concentrations were measured.

**Results:**

Only PO lactose caused a significant increase in blood glucose concentration (*P* < .05). All IV glucose doses induced hyperglycemia and hyperinsulinemia. Concentrations of GIP and GLP‐1 decreased until foals nursed (*P* < .05), at which time rapid increases in glucose, insulin, GIP, and GLP‐1 concentrations occurred (*P* < .05).

**Conclusions and Clinical Importance:**

Healthy newborn foals have a functional EIA that is more responsive to milk and lactose than glucose. Non‐carbohydrate factors in mare's milk may be important for EIA activity. Constant exposure of intestinal cells to nutrients to maintain EIA activity could be relevant to management of sick foals. Foals can be fasted for 4 hours without experiencing hypoglycemia.

**Abbreviations**
aGLP‐1: active glucagon‐like peptide‐1
AUC: area under the curve
DPP‐4: dipeptidyl peptidase 4
EDTA: ethylenediaminetetraacetic acid
EIA: enteroinsular axis
GIP: glucose‐dependent insulinotropic polypeptide
GLP‐1: glucagon‐like peptide‐1
IgG: immunoglobulin G
IVGT: IV glucose test
OGT: oral glucose test
OLT: oral lactose test

## 1 INTRODUCTION

The enteroinsular axis (EIA) comprises intestinal factors (incretins) that stimulate insulin release after PO ingestion of nutrients. Glucose‐dependent insulinotropic polypeptide (GIP) and glucagon‐like peptide‐1 (GLP‐1) are the main incretins. Incretins are secreted by enteroendocrine cells (GIP by K cells in the small intestine; GLP‐1 by L cells in the distal small intestine and colon) in response to the PO intake of nutrients (carbohydrates, fats, and amino acids). They bind to their respective cell membrane receptors in pancreatic β‐cells to enhance insulin secretion and facilitate glucose disposal.^1^, ^2^, ^3^, ^4^ In humans, it has been estimated that GIP and GLP‐1 account for 50%‐70% of insulin secretion after ingestion of a meal.^5^ Incretins also inhibit pancreatic glucagon and somatostatin secretion, maintain β‐cell mass, delay gastric emptying, and promote satiety.^1^, ^2^, ^3^, ^6^, ^7^, ^8^, ^9^

Insulin is considered a central hormone for energy regulation. It increases cellular glucose uptake, stimulates glycogenesis, promotes lipogenesis, and decreases lipolysis and proteolysis. Although blood glucose is a major stimulus for insulin release in the horse, GLP‐1 and GIP contribute to the overall insulin response in healthy and insulin‐dysregulated ponies and horses after ingestion of soluble carbohydrates.^10^, ^11^, ^12^, ^13^, ^14^ A strong association between active GLP‐1 (aGLP‐1) and insulin response to PO nonstructural carbohydrates was documented in ponies.^10^ The same study indicated that 22.7% of the variation in insulin concentrations was attributable to variations in aGLP‐1 concentrations.^10^

Problems of energy dysregulation are frequent in critically ill foals, with up to 70% of foals presenting to a neonatal intensive care unit having a blood glucose concentration outside of the reference range.^15^ Both hypoglycemia and hyperglycemia have been associated with nonsurvival in critically ill equine neonates.^15^, ^16^, ^17^ A recent study in critically ill foals found that hypoglycemia, hypertriglyceridemia, as well as increased glucagon and decreased insulin concentrations were common findings.^16^ Dysregulation of various endocrine factors that contribute to energy metabolism, including growth hormone, ghrelin, insulin‐like growth factor‐1, adrenocorticotropic hormone, cortisol, leptin, and thyroid hormones has been documented in hospitalized foals.^15^, ^18^, ^19^, ^20^, ^21^ Information describing gastrointestinal endocrine factors that regulate metabolic activity in healthy and sick equine neonates is minimal but relevant based on recent information on the importance of the EIA in human patients suffering from type 2 diabetes mellitus^1^, ^2^, ^3^, ^22^ and insulin dysregulation in adult horses.^10^, ^11^, ^23^

Considering that energy disturbances and gastrointestinal disorders are common in critically ill foals, but information on intestinal factors that regulate pancreatic endocrine function is lacking, our goal was to investigate the response of the EIA (GIP, GLP‐1, and insulin) in healthy newborn foals exposed to both PO and IV glucose, PO lactose, and fasting. We hypothesized that incretin secretion in response to PO carbohydrates would be higher than the same dose of glucose administered IV or when compared to a similar period of fasting. We also hypothesized that this incretin response would be linked proportionately to insulin concentrations. Understanding the biology of the EIA in healthy foals could have clinical implications in the management of foals with energy dysregulation and gastrointestinal disease.

## 2 MATERIALS AND METHODS

### 2.1 Experimental design

Thirty‐six Standardbred foals ≤4 days of age, owned by a private breeding farm, were included in this prospective, randomized study. Foals were considered healthy based on physical examination findings, normal CBC, and serum immunoglobulin G (IgG) concentration (>800 mg/dL). Testing took place over two 6‐week periods from March to April during the 2017 (n = 17) and 2018 (n = 19) foaling seasons. Each foal was randomly assigned to the dextrose, lactose, or fasted experimental group. Foals receiving dextrose (n = 24) were randomly assigned to PO or IV route of administration of a low, medium, or high dose in a crossover design, with the alternative route of administration occurring the next day. Foals in the lactose and fasted groups were only sampled on day 1. Care was taken to minimize stress on these foals. Foals remained confined to stalls with their mares between sampling times.

At 60 minutes before initiation of the experimental period, foals were muzzled and an IV catheter (SURFLO EFTE IV Catheter 14G × 2″, Terumo Medical Corp, Somerset, New Jersey) was placed in the jugular vein using local anesthesia. Foals were manually restrained, and no sedative medications were administered at any point. Foals were muzzled for 240 minutes (−60 to 180 minutes).

This study was approved by the Ohio State University Institutional Animal Care and Use Committee and adhered to the principles of humane treatment of animals in veterinary clinical investigations as stated by the American College of Veterinary Internal Medicine and National Institute of Health guidelines.

### 2.2 Oral glucose test (OGT)

Foals were given a 50% dextrose solution (VetOne, MWI Animal Health, Boise, Idaho) at the preassigned dosages: 300 mg/kg (n = 12; OGT‐300), 500 mg/kg (n = 6; OGT‐500), or 1000 mg/kg (n = 6; OGT‐1000). The dextrose was administered PO using a 60‐mL catheter‐tip syringe over a period of 1 minute. Care was taken to minimize spillage.

### 2.3 Intravenous glucose test (IVGT)

Foals were given a 50% dextrose solution (VetOne, MWI Animal Health) through an IV catheter at the preassigned dosages: 300 mg/kg (n = 9; IVGT‐300), 500 mg/kg (n = 5; IVGT‐500), or 1000 mg/kg (n = 5; IVGT‐1000). Administration occurred over a period of 1 minute and the IV catheter was irrigated with 20 mL heparinized saline after administration.

### 2.4 Oral lactose test (OLT)

Foals given lactose (Millipore Sigma, St. Louis, Missouri) as a 20% solution in water at a dosage of 1000 mg/kg (n = 6) by nasogastric intubation over 1 minute.

### 2.5 Fasted group

A group of foals (n = 6) did not receive any sugar enterally or parenterally and remained muzzled and unable to nurse for the duration of the study (240 minutes).

### 2.6 Blood sampling

Blood samples were collected at time 0 (before carbohydrate administration) and at 5, 10, 15, 30, 45, 60, 90, 120, 150, and 180 minutes. After 180 minutes, all foals were unmuzzled, allowed to nurse from the mare ad libitum, and additional blood samples in most (n = 22) foals were collected at 195 and 210 minutes.

Blood samples (4 mL) were placed in prechilled ethylenediaminetetraacetic acid (EDTA) tubes containing aprotinin (GoldBio, St Louis, Missouri) and diprotin A (Bachem, Torrance, California). Aprotinin (500 kU/mL of blood) was added to inhibit protease‐mediated degradation of peptide hormones and diprotin A (50 μmol/mL of blood) is a dipeptidyl peptidase‐4 (DPP‐4) protease inhibitor added to decrease degradation of GIP and GLP‐1. The EDTA‐aprotinin‐DPP‐4 inhibitor tubes immediately were placed on ice for at least 20 minutes. Tubes were centrifuged at 1000*g* for 10 minutes at 4°C. Within 6 hours of collection, plasma was aliquoted and stored at −80°C until analysis. Blood samples for CBC and IgG concentrations were processed within 4 hours of collection.

### 2.7 Sample analysis

Blood glucose concentrations were measured immediately after collection using a portable glucometer (AlphaTRAK 2 blood glucose monitoring system, Zoetis, Parsippany, New Jersey) previously validated for horses.^24^ Commercially available ELISA kits previously validated for horses were used to measure plasma total GIP (EZHGIP‐54K, Millipore Sigma) and plasma total GLP‐1 (EZGLP1T‐36 K, Millipore Sigma) concentrations.^10^ Plasma insulin concentrations were measured using a human‐specific ELISA (07M‐60102, MP Biomedicals, Solon, Ohio) that had linearity at dilutions up to 1:8, inter‐ and intra‐assay coefficients of variation of <10% for equine samples, a working range of 1‐300 μIU/mL, and a detection limit of 0.75 μIU/mL.

### 2.8 Data analysis

Data sets were tested for normality using the Shapiro‐Wilk normality test and were not normally distributed. Therefore, median and interquartile ranges were calculated. Comparisons among groups were carried out with the Kruskal‐Wallis statistic, and Dunn's post hoc test was used to compare each time point individually within the group. Comparisons over time were performed using the Friedman test. Peak concentrations were measured for each foal (Cmax) and areas under the curve (AUCs) for glucose (glucose‐AUC), insulin (insulin‐AUC), GIP (GIP‐AUC), and GLP‐1 (GLP‐1‐AUC) were calculated using the nonoverlapping trapezoid method. Results are presented as values relative to time 0 and absolute values. Statistical analysis was performed using commercial statistical software (Prism 8.0, GraphPad Software Inc, San Diego, California; SigmaPlot 14, Systat Software, Chicago, Illinois). Statistical significance was set at *P* < .05.

## 3 RESULTS

### 3.1 Study population

Thirty‐six Standardbred foals ≤4 days of age were used. The median age of foals at the time of study participation was 24 hours (range, 8‐96 hours). Fourteen foals <24 hours of age were included in the study. Three each were used in the 500 mg/kg, 1000 mg/kg and lactose study groups, whereas only 1 foal <24 hours of age was included in the 300 mg/kg group and 4 foals were included in the fasted group. The median IgG concentration was 1700 mg/dL (range, 1295‐1957 mg/dL). Twenty‐three of 36 foals (64%) were fillies and 13/36 (36%) were colts.

### 3.2 Blood glucose

**Figure 1:**
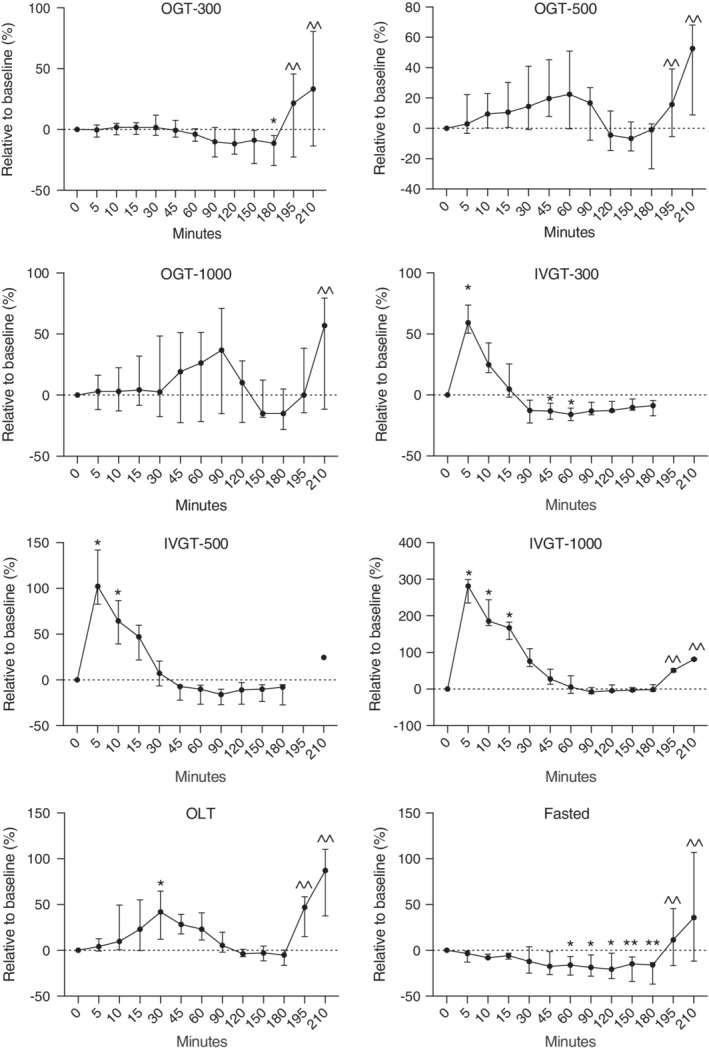
Relative changes in blood glucose concentrations in healthy newborn foals administered glucose orally (300, 500, and 1000 mg/kg) and IV (300, 500, and 1000 mg/kg), lactose (1000 mg/kg), and fasted. Fasted, no treatment; IVGT, IV glucose test; OGT, oral glucose test; OLT, oral lactose test. Values (median, interquartile range [IQR]) presented relative (%) to time 0. Y axis scaling between figures is different to emphasize changes. *P < .05; **P < .01 compared to time 0; ^^*P* < .05 compared to 180 minutes

Median baseline blood glucose concentration for all foals was 140 mg/dL (range, 123‐160 mg/dL; Table 1). The PO administration of 300, 500, or 1000 mg/kg of dextrose did not induce a significant increase in blood glucose concentration at any time point during the testing period when compared to baseline results. In the OGT‐300 group, median glucose concentration at 180 minutes was significantly lower than at time 0 (*P* < .01). Foals in the IVGT‐300, IVGT‐500, and IVGT‐1000 groups had significant increases in blood glucose concentrations at 5‐15 minutes compared to time 0 (Figure 1 and Table 1; *P* < .01). Foals in the OLT group had a significant increase in blood glucose concentration at 30 minutes compared to time 0 (Figure 1; *P* < .05). Fasted foals had a significant decrease in blood glucose concentration from 60 to 180 minutes (*P* < .05). However, glucose concentrations remained within the reference range, foals were active, and no foal showed signs of hypoglycemia. After foals were allowed to nurse, a significant increase in glucose concentration was evident within 15 minutes (between 195 and 210 minutes; Figure 1; *P* < .05).

**Table 1:** Median and interquartile range (IQR) data for blood glucose and insulin in healthy newborn foals administered glucose orally (300, 500, and 1000 mg/kg) and IV (300, 500, and 1000 mg/kg), lactose (1000 mg/kg), and fasted

Group/Time	0 min	5 min	30 min	120 min	180 min	210 min
Glucose (mg/dL)						
OGT‐300	159.5 (153.5‐183.8)	166 (150.3‐181.0)	174.5 (151.5‐179.3)	150 (135‐160)	140 (136‐146.5)**	192 (147‐251)
OGT‐500	120.5 (97‐169.5)	124(113‐169)	138.5 (133.5‐171.3)	116.5 (105.3‐142.8)	115.5 (85.8‐151)	123 (116‐232)
OGT‐1000	135 (116.3‐159.8)	134.5 (126‐159.3)	152 (122‐179)	148 (135.3‐155.5)	110 (97‐166)	210 (183‐226)^
IVGT‐300	150 (135.5‐180.5)	240 (235‐266.5)**	131 (118.5‐151.5)	139 (121.5‐154.5)	138 (122‐149)	‐
IVGT‐500	138 (130‐172.5)	296 (262‐362.5)**	168 (132‐179)	121 (119‐133)	121 (114‐136)	172
IVGT‐1000	118 (105.5‐130.5)	432 (408‐459.5)**	195 (189.5‐254.5)	114 (106.5‐133)	120 (114.5‐124.5)	212 (196‐224)^
OLT	126.5 (119.8‐161.5)	140 (131.8‐160)	197.5 (163.8‐207.5)*	119.5 (116.8‐165)	119.0 (113‐131)	238 (214.3‐255.5)^
Fasted	139.5 (125‐145.3)	128 (118‐137.5)	115 (104.5‐145.8)	112.5 (91.8‐119.3)*	108 (83.8‐122)**	191.5 (125‐253.5)^
Insulin (μIU/mL)						
OGT‐300	9.75 (5.9‐19.4)	11.0 (6.0‐19.2)	13.2 (9‐21.2)	6.3 (2.4‐10.3)**	6.1 (2.7‐10.7)	31.6 (15.3‐64.0)^
OGT‐500	14.4 (8.5‐26.5)	9.6 (4.2‐19.0)	12.5 (7.5‐19.9)	6.0 (2.3‐21.7)	6.2 (1.6‐15.2)	13.9 (4.9‐43.10)
OGT‐1000	7.6 (3.0‐23.2)	11.2 (3.8‐21.7)	8.8 (4.1‐27.3)	5.6 (2.3‐8.4)	5.5 (1.2‐19.8)	59.5 (31.3‐102.1)^
IVGT‐300	6.0 (3.7‐13.5)	51.3 (35.5‐56.7)*	5.9 (4.4‐10.2)	4.0 (2.8‐9.7)	6.9 (1.5‐10.1)	‐
IVGT‐500	14.7 (7.6‐23.3)	60.2 (54.8‐96.9)*	11.7 (7.8‐22.3)	7.0 (2.4‐10.3)	4.7 (2.2‐11.7)	23.2
IVGT‐1000	2.4 (1.0‐10.0)	78.5 (51.8‐166.2)**	33.2 (12.2‐67.2)	4.2 (2.0‐7.1)	3.4 (0.9‐6.8)	72.2 (30.7‐140.2)^
OLT	11.8 (9.8‐31.5)	31.4 (12.1‐58.3)	38.1 (26.0‐51.5)	8.7 (5.9‐16.5)	12.4 (8.4‐13.3)	66.3 (25.6‐115.8)^
Fasted	18.3 (10.4‐53.5)	13.9 (8.5‐46.4)	7.6 (5.7‐25.2)	7.3 (3.5‐10.4)**	6.1 (3.5‐7.6)**	48.6 (23.7‐85.9)^^

Abbreviations: Fasted, no oral or IV treatment; IVGT‐300, 300 mg/kg of glucose; IVGT‐500, 500 mg/kg of glucose; IVGT‐1000, 1000 mg/kg of glucose; OGT‐300, 300 mg/kg of glucose; OGT‐500, 500 mg/kg of glucose; OGT‐1000, 1000 mg/kg of glucose; OLT, 1000 mg/kg of lactose.

*P < .05, **P < .01 compared to time 0; ^*P* < .05, ^^*P* < .01 compared to time 180 minutes.

When changes in blood glucose concentrations were adjusted to baseline results, the maximum concentration (which was observed at variable time points) increased by 1.8, 22.5, 36.8, and 41.9% over baseline for the OGT‐300, OGT‐500, OGT‐1000, and OLT groups, respectively (Figure 1). For the IVGT‐300, IVT‐500, and IVGT‐1000 groups, blood glucose concentrations increased by 59.2, 102.2, and 284.4%, respectively (Figure 1; *P* < .05). Compared to baseline results, blood glucose concentrations in fasted foals decreased by 16% at 180 minutes (*P* < .05).

### 3.3 Insulin

**Figure 2:**
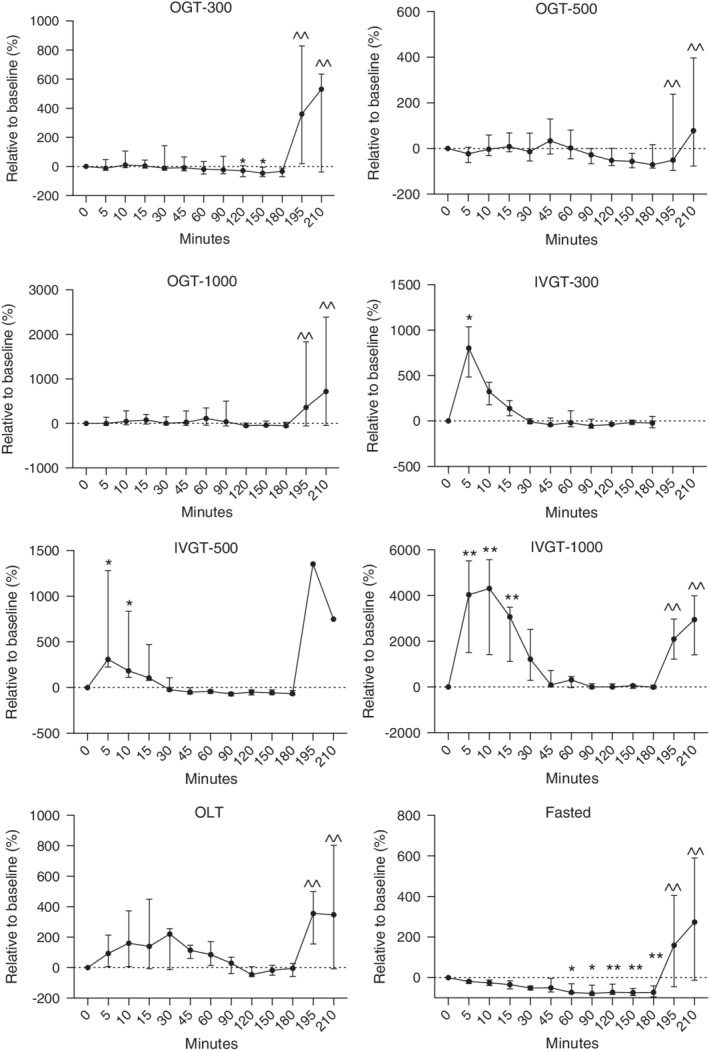
Relative changes in plasma insulin concentrations in healthy newborn foals administered glucose orally (300, 500, and 1000 mg/kg) and IV (300, 500, and 1000 mg/kg), lactose (1000 mg/kg), and fasted. Fasted, no treatment; IVGT, IV glucose test; OGT, oral glucose test; OLT, oral lactose test. Values (median, interquartile range [IQR]) presented relative (%) to time 0. Y axis scaling between figures is different to emphasize changes. *P < .05; **P < .01 compared to time 0; ^^*P* < .05 compared to 180 minutes

Baseline plasma insulin concentration for all foals was 10.7 μIU/mL (range, 6.0‐16.70 μIU/mL; Table 1). No statistically significant increase in plasma insulin concentrations from baseline concentrations occurred during the OGT‐300, OGT‐500, OGT‐1000, or OLT (Figure 2). However, higher insulin concentrations were noted between 10 and 60 minutes for the OGT (all doses) and OLT groups. A significant decrease in insulin concentrations was noted for the OGT‐300 group between 120 and 150 minutes and the fasted group between 60 and 180 minutes (*P* < .05). The IV administration of dextrose produced significant increases in insulin concentrations between 5 and 15 minutes compared to time 0 (*P* < .05).

As a relative percentage change from baseline, insulin concentrations increased by 10.7, 33.4, 110.6, and 220.3% for the OGT‐300, OGT‐500, OGT‐1000, and OLT groups, respectively (Figure 2; *P* < .05). For the IVGT‐300, IVT‐500, and IVGT‐1000 groups, insulin concentrations increased by 802, 309.9, and 4238%, respectively (Figure 2; *P* < .05). Compared to baseline results, insulin concentrations in fasted foals decreased by 72.5% at 180 minutes.

### 3.4 Glucose‐dependent insulinotropic polypeptide (GIP)

**Figure 3:**
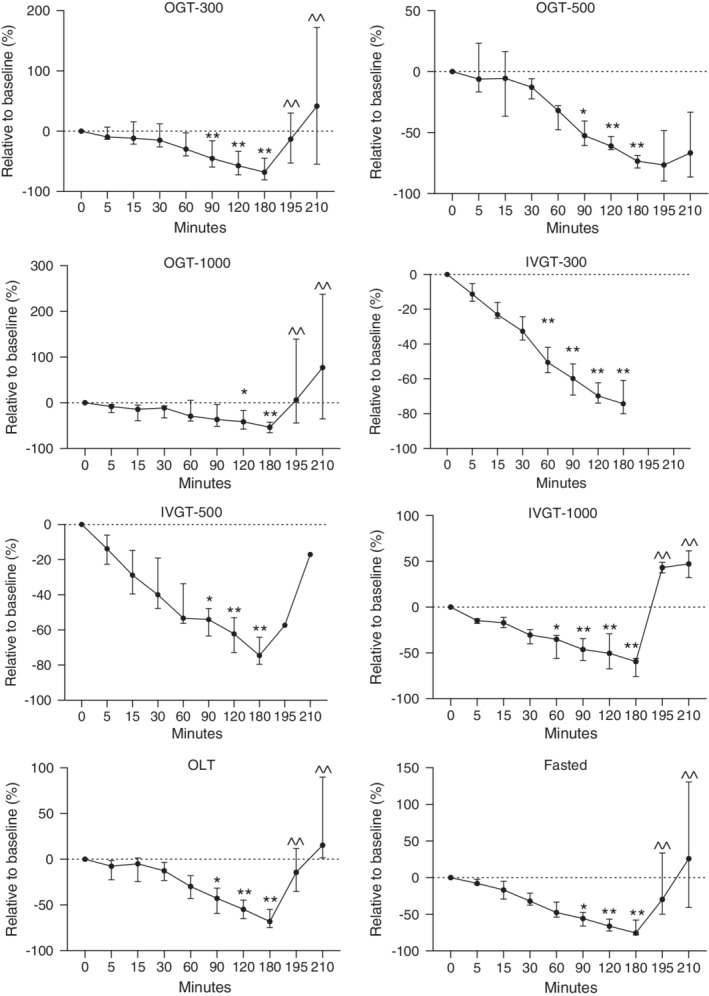
Relative changes in plasma GIP concentrations in healthy newborn foals administered glucose orally (300, 500, and 1000 mg/kg) and IV (300, 500, and 1000 mg/kg), lactose (1000 mg/kg), and fasted. Fasted, no treatment; IVGT, IV glucose test; OGT, oral glucose test; OLT, oral lactose test. Values (median, interquartile range [IQR]) presented relative (%) to time 0. Y axis scaling between figures is different to emphasize changes. *P < .05; **P < .01 compared to time 0; ^^*P* < .05 compared to 180 minutes

Baseline GIP concentration for all foals was 238.2 pg/mL (range, 137.4‐391.3 pg/mL; Table 2). From baseline, plasma GIP concentrations decreased significantly from 90 to 180 minutes for the OGT‐300 and OGT‐500 groups and from 120 to 180 minutes for the OGT‐1000 group (Figure 3; *P* < .05). In the IVGT‐300, IVGT‐500, and IVGT‐1000 groups, GIP concentrations decreased earlier and longer (60‐180 minutes; Figure 3; *P* < .05).

**Table 2:** Median and interquartile range (IQR) data for GIP and GLP‐1 in healthy newborn foals administered glucose orally (300, 500, and 1000 mg/kg) and IV (300, 500, and 1000 mg/kg), lactose (1000 mg/kg), and fasted

Group/Time	0 min	5 min	30 min	120 min	180 min	210 min
GIP (pg/mL)						
OGT‐300	247 (94.8‐345.2)	232.8 (121.8‐337.8)	210.5 (120.2‐344.6)	92.9 (48.7‐180.1)**	90.6 (34.6‐206.6)**	219.1 (194.5‐584.3)
OGT‐500	707.4 (374.1‐1041.0)	741.0 (335.6‐1084)	670.8 (302.5‐917.4)	313.3 (158.4‐404.1)**	167.8 (121.0‐270.9)**	280.2 (116‐282.5)
OGT‐1000	229.7 (151.8‐558.6)	203.1 (100.3‐524.4)	174.2 (99.8‐523.9)	136.5 (72.8‐345.3)*	96.6 (51.2‐278.3)**	317.1 (236.6‐715)
IVGT‐300	130.1 (97.7‐239.5)	117.0 (88.4‐210.0)	80.7 (65.9‐194.7)	37.3 (29.3‐68.0)**	30.6 (25.1‐49.7)**	‐
IVGT‐500	247.1 (157.4‐344.4)	217.1 (129.3‐291.6)	161.7 (79.1‐256.1)	78.1 (47.0‐147.1)**	57.4 (36.8‐104.4)**	211.1
IVGT‐1000	137.6 (103.2‐144.6)	110.5 (87.7‐125.7)	77.1 (70.7‐105.4)	45.5 (40.1‐102.6)**	37.3 (30.2‐63.9)**	238.8 (216.7‐269.4)^
OLT	311.0 (205.5‐645.6)	279.2 (201.5‐482.3)	289 (183.6‐440.2)	144.8 (102.1‐277.4)**	108.1 (77.7‐209.3)**	503.6 (233.8‐824.6)^^
Fasted	366.6 (169.4‐1066)	339 (152.7‐1052)	249.3 (105.7‐868.6)	124.5 (43.7‐462.2)**	108.2 (39.0‐398.1)**	369.5 (190.3‐795.6)
GLP‐1 (pM)						
OGT‐300	137.8 (114.2‐192.7)	150.1 (126.5‐269.1)	164.8 (105‐269.6)	125.9 (83.2‐209.4)**	143.3 (88.2‐219.6)	171.2 (149.8‐320.9)
OGT‐500	157.4 (89.1‐295.4)	151.1 (67.1‐354.9)	142.4 (50.3‐317.7)	120.7 (44.6‐301.0)	90.3 (41.7‐166.9)**	101.0 (23.3‐274.0)
OGT‐1000	49.6 (28.3‐156.1)	71.1 (48.1‐169.8)	68.1 (48.8‐163.2)	43.7 (24.3‐124.6)	38.8 (30.4‐113.5)	62.5 (37.8‐232.1)
IVGT‐300	99.1 (67.8‐145.2)	82.3 (60.9‐136.3)	67.6 (49.0‐133.3)	58.6 (48.1‐114.2)**	71.3 (53.5‐122.0)	‐
IVGT‐500	129.7 (85.7‐318)	111.8 (78.7‐223.1)	82.0 (52.0‐277.1)*	57.2 (52.3‐229)**	58.7 (54.1‐246.6)*	121.6
IVGT‐1000	51.5 (31.8‐179.3)	47.0 (25.9‐163.7)	68.1 (21.8‐162.1)	46.3 (21.3‐180.9)	40.6 (22.6‐139.1)	70.5 (59.8‐155.7)
OLT	67.1 (39.5‐114.2)	70.0 (39.5‐105.1)	57.5 (35.7‐102.9)	37.2 (24.1‐79.8)**	37.2 (27.3‐73.4)**	67.4 (45.8‐119.7)
Fasted	97 (58.3‐157.3)	97.4 (66.2‐162.2)	97.3 (52.7‐151.9)	83.0 (39.5‐151.0)	81.6 (49.6‐154.3)	119.7 (98.6‐172.6)

Abbreviations: Fasted, no oral or IV treatment; GIP, glucose‐dependent insulinotropic polypeptide; GLP‐1, glucagon‐like peptide‐1; IVGT‐300, 300 mg/kg of glucose; IVGT‐500, 500 mg/kg of glucose; IVGT‐1000, 1000 mg/kg of glucose; OGT‐300, 300 mg/kg of glucose; OGT‐500, 500 mg/kg of glucose; OGT‐1000, 1000 mg/kg of glucose; OLT, 1000 mg/kg of lactose.

*P < .05, **P < .01 compared to time 0; ^*P* < .05, ^^*P* < .01 compared to time 180 minutes.

As a relative percentage change from baseline, GIP concentrations decreased by 67.83, 73.4, 53.4, and 68% for the OGT‐300, OGT‐500, OGT‐1000, and OLT groups, respectively, at 180 minutes (Figure 3; *P* < .05). For the IVGT‐300, IVGT‐500, and IVGT‐1000 groups, GIP concentrations decreased by 74.3, 74.6, and 59.4%, respectively (Figure 3; *P* < .05), at 180 minutes. Compared to baseline, GIP concentrations in fasted foals decreased by 75.5% at 180 minutes.

### 3.5 Glucagon‐like peptide 1 (GLP‐1)

**Figure 4:**
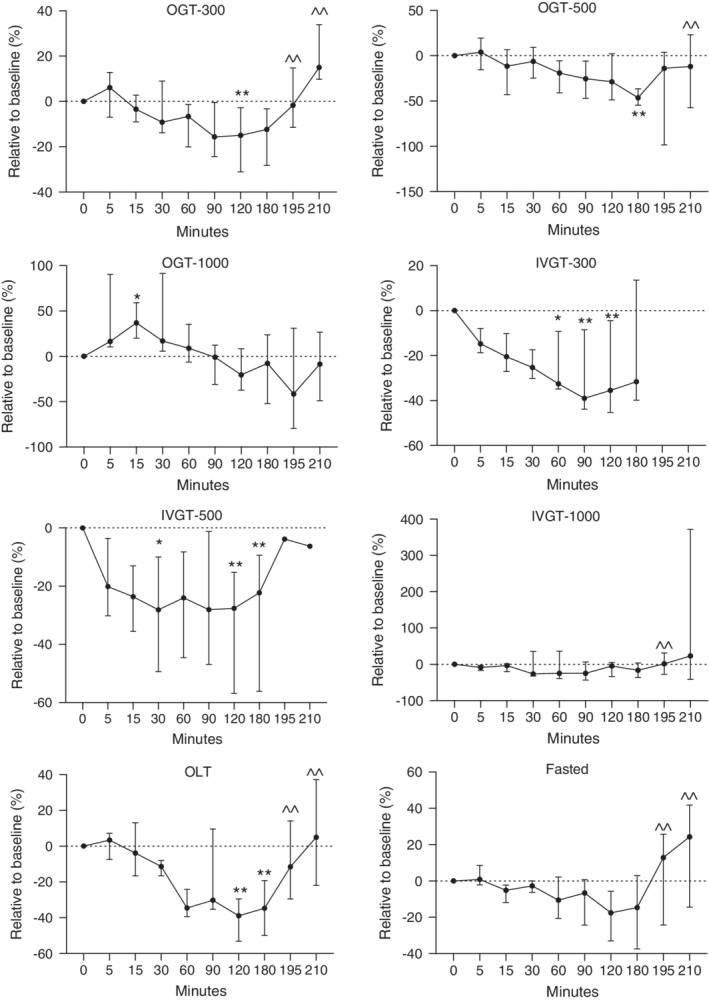
Relative changes in plasma GLP‐1 concentrations in healthy newborn foals administered glucose orally (300, 500, and 1000 mg/kg) and IV (300, 500, and 1000 mg/kg), lactose (1000 mg/kg), and fasted. Fasted, no treatment; IVGT, IV glucose test; OGT, oral glucose test; OLT, oral lactose test. Values (median, interquartile range [IQR]) presented relative (%) to time 0. Y axis scaling between figures is different to emphasize changes. *P < .05; **P < .01 compared to time 0; ^^*P* < .05 compared to 180 minutes

Baseline GLP‐1 for all foals was 113 pM (range, 57.30‐173.5 pM; Table 2). The only group that experienced a statistically significant increase in GLP‐1 was the OGT‐1000 at 15 minutes (*P* < .05). Plasma GLP‐1 concentrations showed a statistically significant decrease in the OGT‐300 (120 minutes) and OGT‐500 (180 minutes) groups (*P* < .05). Plasma GLP‐1 concentrations also decreased in the IVGT‐300 and IVGT‐500 groups from 30 to 180 minutes (Figure 4; *P* < .05). Foals in the OLT group had significantly lower GLP‐1 concentrations from 120 to 180 minutes (Figure 4). In the fasted and IVGT‐1000 groups, no significant change occurred in GLP‐1 concentrations throughout the testing period.

**Figure 5:**
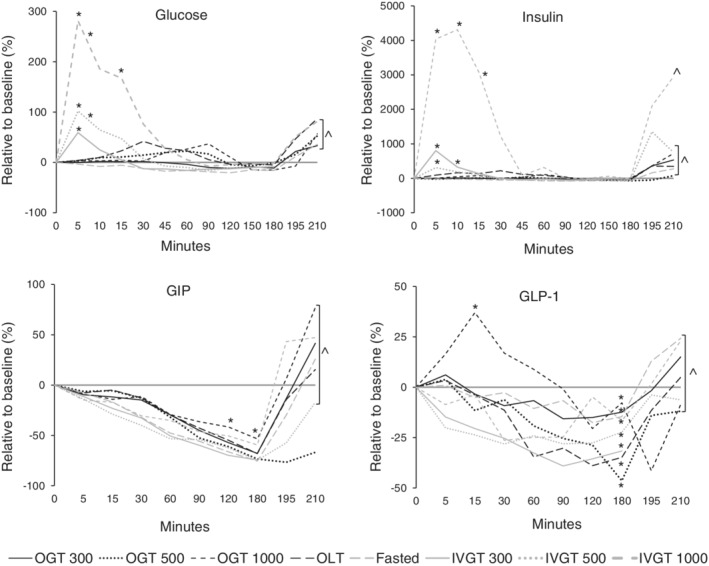
Relative changes in blood glucose, insulin, GLP‐1, and GIP concentrations in healthy newborn foals. Fasted, no treatment; IVGT, IV glucose test; OGT, oral glucose test; OLT, oral lactose test. Values presented relative (%) to time 0. interquartile range [IQR] and statistical significance have been omitted but presented in Figures 1, 2, 3, 4

As a relative percentage change from baseline, GLP‐1 concentrations decreased by 12.4, 46.38, 7.8, and 34.8% for the OGT‐300, OGT‐500, OGT‐1000, and OLT groups, respectively, at 180 minutes (Figure 4; *P* < .05). For the IVGT‐300, IVT‐500, and IVGT‐1000 groups, GLP‐1 concentrations decreased by 31.6, 22.3, and 16.6%, respectively (Figure 4; *P* < .05), at 180 minutes. Compared to baseline, GLP‐1 concentrations in fasted foals decreased by 14.6% at 180 minutes. Figure 5 provides an overall picture of the relative changes in glucose, insulin, GIP, and GLP‐1 concentrations.

### 3.6 Area under the curve for glucose, plasma insulin, plasma GIP, and plasma GLP‐1

The glucose‐AUC and GIP‐AUC were statistically different among study groups (Table 3). Foals in the OGT‐300, OGT‐1000, and IVGT‐1000 groups had significantly higher glucose‐AUC compared to fasted foals (*P* < .05). Insulin‐AUC was not different among groups, except for the OLT group, which had a higher AUC compared to the fasted group (*P* < .05). For GIP, the AUC was not different between treated and fasted foals, but, when comparing equivalent PO and IV doses, the AUC for PO glucose was larger (*P* < .05). No significant difference for GIP‐AUC was found between fasted foals and the other study groups.

**Table 3:** Median and interquartile range (IQR) data for area under the curve (AUC) for glucose (AUC‐glucose), insulin (AUC‐insulin), GIP (AUC‐GIP), and GLP‐1 (AUC‐GLP‐1) in healthy newborn foals administered glucose orally (300, 500, and 1000 mg/kg) and IV (300, 500, and 1000 mg/kg), lactose (1000 mg/kg)

	Glucose (mg*min/dL)	Insulin (μIU*min/mL)	GIP (pg*min/mL)	GLP‐1 (pmol*min/L)
OGT‐300	461.5 (418.7‐497.9)**	29.1 (17.3‐35.1)	466.9 (227.1‐723.7)	402.8 (291.7‐698.6)
OGT‐500	379.5 (347.2‐493.8)	32.6 (24.6‐51.7)	1285 (616.9‐1771)	373.6 (133.2‐798.8)
OGT‐1000	453 (402‐520.8)	32.7 (23.7‐43.8)	433 (247.2‐1204)	161.6 (100.9‐421.2)
IVGT‐300	444.9 (375.7‐462)*	24.2 (15.8‐45.4)	157.9 (142.1‐350.2)	199.3 (154.4‐385.4)
IVGT‐500	419 (383.1‐460.6)	31.3 (21.3‐53.2)	340.5 (194.6‐545.3)	223.2 (171.9‐803)
IVGT‐1000	439.4 (430.9‐517.1)*	42 (21.8‐86.4)	167 (164.8‐292.9)	175.5 (65‐486.1)
OLT	417.5 (404.8‐527.3)	57.2 (50.5‐87.2)*	577.9 (414.7‐998.7)	138.8 (91.4‐255)
Fasted	344.2 (300.9‐376.4)	25.2 (16‐44.6)	547.7 (215.3‐1912)	266.4 (143.7‐460.1)

Abbreviations: Fasted, no oral or IV treatment; GIP, glucose‐dependent insulinotropic polypeptide; GLP‐1, glucagon‐like peptide‐1; IVGT‐300, 300 mg/kg of glucose; IVGT‐500, 500 mg/kg of glucose; IVGT‐1000, 1000 mg/kg of glucose; OGT‐300, 300 mg/kg of glucose; OGT‐500, 500 mg/kg of glucose; OGT‐1000, 1000 mg/kg of glucose; OLT, 1000 mg/kg of lactose.

*P < .05, compared to fasted foals; **P < .01 compared to fasted foals.

### 3.7 Access to free choice nursing

**Figure 6:**
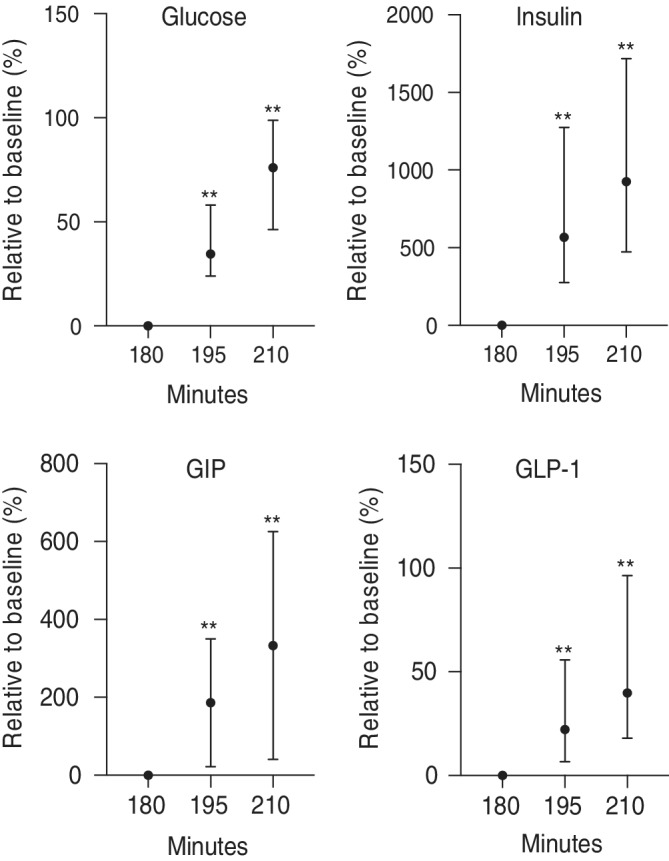
Relative changes in blood glucose, insulin, GIP, and GLP‐1 concentrations in all newborn foals after allowed to nurse. Values presented relative (%) to 180 minutes. **P < .01 compared to 180 minutes

Access to free choice nursing resulted in a rapid and significant increase in blood glucose (34.6% at 195 minutes and 76% at 210 minutes), insulin (551% at 195 minutes and 637% at 210 minutes), GIP (137% at 195 minutes and 268% at 210 minutes), and GLP‐1 (21% at 195 minutes and 39.8% at 210 minutes) concentrations compared to 180 minutes (Figure 6; *P* < .05). These increases were larger than those seen after PO oral dextrose or lactose administration. The increase in GIP concentrations was more evident than for GLP‐1 concentrations.

## 4 DISCUSSION

In our study, using PO and IV dextrose, PO lactose, and fasting, we documented that healthy equine neonates have a functional EIA. We also found that the response of the EIA in equine neonates in the immediate postpartum period is highly variable. To our knowledge, ours is the first study to investigate the EIA in healthy newborn foals.

Although the EIA response to lactose was the most evident, we were surprised with the minimal increases in GIP, GLP‐1, and insulin concentrations despite high PO carbohydrate doses. This is a novel finding in equine neonatal and comparative endocrinology that deserves further investigation. For horses and ponies, PO glucose doses of 150 mg/kg are considered appropriate to assess pancreatic insulin secretion,^25^, ^26^, ^27^ and in general higher doses are unnecessary. Incretin and insulin concentrations in the foals of our study followed a similar pattern. Despite high PO glucose and lactose dosing, both GLP‐1 and GIP concentrations continued to decrease until foals were allowed to nurse. The minimal incretin response to enteric carbohydrates is the most likely explanation for the negligible to absent insulin secretion in these foals. This hypothesis is further supported by marginal changes in glycemia with PO glucose and lactose administration; however, IV glucose administration induced rapid and significant insulin secretion.

The rapid insulin increase without an incretin response after IV dextrose administration provides additional validation to the functionality of the EIA in equine neonates and is consistent with similar findings in ponies, where enteral glucose provoked GIP and GLP‐1 secretion, but IV administration did not.^10^, ^14^

In contrast to the minimal incretin response to PO glucose or lactose, once foals were allowed to nurse, a rapid and significant increase in both GIP and GLP‐1 concentrations occurred over their respective results at baseline and at 180 minutes. This finding indicates that the EIA in newborn foals is functional and highly responsive to nutrients contained in mare's milk, other than those administered in our study. Human newborn infants showed a similar GLP‐1 response after feeding milk.^28^ Mare's milk 1‐4 weeks postpartum is composed of approximately 2% fat, 3% protein, and 6% lactose.^29^ Lactose is a disaccharide composed of glucose and galactose in equal parts. Thus, foals in the lactose group (1000 mg/kg) received an equivalent glucose dose as did foals in the OGT‐500 group. The difference noted between these 2 study groups would be 500 mg/kg of galactose or another undetermined variable. Fat‐based diets in adult ponies previously have been demonstrated to stimulate GIP secretion after a PO glucose test when compared to ponies that had the same testing performed and were maintained on a carbohydrate‐based diet.^30^

Despite glucose, insulin, GIP, and GLP‐1 following a similar trend after nursing, we noticed that compared to 180 minutes, the GIP response at 210 minutes was stronger (approximately 300%) than for GLP‐1 (approximately 40%). Potential explanations for this increase could be that GLP‐1 is degraded faster by DPP‐4, that GIP is stimulated differently by various dietary components as compared to GLP‐1 as previously demonstrated in humans, rats, and swine^3^, ^31^, ^32^, ^33^ or that GIP is a more important incretin in the early neonatal period.

Incretin and insulin dynamic changes may be different in older foals and may represent transition from in utero to extrauterine life. Maturation of the energy endocrine axis in foals continues in the postpartum period. Foals are inherently insulin resistant in the first days after birth and this finding has been attributed to activation of the hypothalamic‐pituitary‐adrenal axis and increased cortisol concentrations,^34^, ^35^, ^36^ which could be a reason for individual variation among the foals in our study. In addition to incretins, other factors that may have contributed to glucose dynamics and insulin secretion in the foals of our study include gastrointestinal hormones (eg, gastrin, secretin, and ghrelin), somatostatin, and glucagon.

Regarding clinical relevance, our research sheds light on the importance of constant exposure of intestinal cells to nutrients to maintain a functional EIA, because impaired incretin secretion can further complicate glucose regulation in critically ill foals. In addition, conditions that damage intestinal epithelial cells such as ischemia and infections (viral or bacterial) can indirectly disturb the endocrine pancreas and energy metabolism. Many foals admitted to equine hospitals with evidence of energy dysregulation may have a dysfunctional EIA that goes unnoticed because these factors are not measured clinically. In support of this hypothesis, we recently found that foals with severe sepsis tend to have lower insulin and GIP concentrations (L.M.R./R.E.T., personal communication).

Another point of interest regarding neonatal endocrinology is the >5‐fold increase in GLP‐1 and GIP concentrations in the foals of our study compared to results reported using the same assays for adult ponies and horses.^10^, ^11^, ^12^, ^13^ This finding indicates that equine enteroendocrine cells have a high capacity to produce incretins in the early neonatal period, perhaps as an evolutionary adaptation to unique components of mare's milk (eg, fatty acids, carbohydrates, and amino acids) and the need for rapid glucose disposal. It could also be that newborn foals have decreased DPP‐4 activity compared to horses. Similar to foals and horses, it has been shown that human infants have higher resting GLP‐1 concentrations than adults.^28^ The dynamics of incretin secretion over time in healthy foals remain to be investigated but preliminary work from our laboratory indicates that 3‐day‐old foals have lower GLP‐1 concentrations than at birth (L.M.R./R.E.T., personal communication).

Limitations of our study include the relatively small sample size considering variations in hormone concentrations in the first 48 hours after birth, different methods of PO carbohydrate administration, as well as the concentrations and volumes of PO solutions used (50% dextrose versus 20% lactose). Pancreatic β‐cell response to glucose is low immediately postpartum compared to foals 5‐7 days of age,^36^, ^37^ whereas the foals of our study were <4 days of age. A narrower age range of foals would have been ideal but foal access was dictated by the farm. It would be valuable to investigate incretin dynamic changes that occur in the first week after birth. The nutritional management of the mares was not considered in our study. Doing so may be warranted in future investigations because high starch diets fed to gestating mares have been shown to influence glucose and insulin dynamics in their offspring.^38^ Considering that under natural conditions foals do not consume pure glucose, in addition to the marked EIA activation observed when foals nursed compared to after PO glucose or lactose, it will be important to evaluate other substrates (eg, amino acids, fats) to better characterize the response of the EIA in equine neonates. Plasma aGLP‐1 (GLP‐1 [7‐36] amide and GLP‐1 [7‐37]), the bioactive form of GLP‐1 that exists before degradation by DPP‐4 into total GLP‐1, was not measured in our foals but should be considered in future studies. In people, only 10%‐15% of aGLP‐1 enters systemic circulation and reaches the pancreas and other organs.^39^ Therefore, measuring total GLP‐1 (aGLP‐1 and metabolites) gives a better estimation of intestinal L‐cell secretion of GLP‐1.^39^ Additionally, quantification of catecholamines and cortisol is warranted in future studies because stress from handling and muzzling potentially could increase these hormones and affect the results of this type of study.

In conclusion, we documented that healthy newborn foals have a functional EIA. Although activation of the EIA was minimal in response to PO glucose and lactose at doses up to 1000 mg/kg, rapid and significant increases in GIP and GLP‐1 concentrations were noted when foals were allowed to resume nursing ad libitum. Future research on the EIA in foals should focus on other substrates that may stimulate incretin release and their potential therapeutic implications.

## CONFLICT OF INTEREST DECLARATION

Authors declare no conflict of interest.

## OFF‐LABEL ANTIMICROBIAL DECLARATION

Authors declare no off‐label use of antimicrobials.

## INSTITUTIONAL ANIMAL CARE AND USE COMMITTEE (IACUC) OR OTHER APPROVAL DECLARATION

Approval from The Ohio State IACUC, number 2008A0170.

## HUMAN ETHICS APPROVAL DECLARATION

Authors declare human ethics approval was not needed for this study.
